# Editorial: Psychological factors in physical education and sport, volume III

**DOI:** 10.3389/fpsyg.2024.1425279

**Published:** 2024-05-15

**Authors:** David Manzano-Sánchez, Carla Chicau Borrego, Marianna Alesi, Manuel Gómez-López

**Affiliations:** ^1^Department of Didactic of Plastic, Musical, and Body Expression, Faculty of Education and Psychology, University of Extremadura, Badajoz, Spain; ^2^Sport Sciences School of Rio Maior, Polytechnic Institute of Santarém, Research Center in Life Quality, Rio Maior, Portugal; ^3^Department of Psychology, Educational Sciences, and Human Movement, University of Palermo, Palermo, Italy; ^4^Department of Physical Activity and Sport, Faculty of Sports Sciences, University of Murcia, Murcia, Spain

**Keywords:** psychology, motivation, sport, education, physical education, psychological wellbeing

This Research Topic has compiled a large part of the scientific evidence that focuses on psychology and its influence on physical education and sports.

Currently, sports psychology and in the field of Physical Education, is being widely studied, especially due to the relevance it has for both the physical and cognitive development of people.

Thus, it is not surprising that studies such as that of Mira et al. conclude that aspects such as social support from influencers (parents, friends, and coaches) can help athletes with disability to improve their resilience and thus their ability to cope. The study by Dong et al. shows how it is necessary in the short and long term to work on the psychological aspect of athletes to avoid the dreaded “burnout” and to do so, variables such as gratitude, alongside a supportive coach-athlete relationship and elevated levels of hope, may play crucial roles in mitigating burnout symptoms in this case with a sample of 483 active team sports athletes. Linked to the previous study, Cao and Lyu conclude that in addition to adequate psychology on the part of athletes, a high capacity for perseverance on the part of instructors is necessary, above all, promoting more autonomous (self-determined) motivation and reducing demotivation and it is also vital to teach personal and cultural values to improve sports commitment. The study by Huard and Lemoyne also takes on special relevance, given that according to their research, proposals and recommendations must be made for coaches in order to promote positive development in players, with sports competition influencing factors such as early specialization or even the position and age of the players, in this case, as ice hockey players.

Another key point of the present Research Topic, once some of the challenges that athletes have at a cognitive level have been addressed, is that not only is it necessary to have adequate cognitive capacity for the present and future of athletes, but the research by Amoroso et al. indicates the special interest in being able to evaluate the ethical behavior and self-control of athletes, specifically with a sample of elite Ultimate Championships players, allowing the self-referencing to improve ethical behavior across divisions and age groups. The study by Shuai et al. indicates the influence of personality on sport performance. Their review in competitive sports shows how the so-called “five models,” made up of the variables of extraversion, agreeableness, conscientiousness, neuroticism, and openness to new experiences, are transcendental in terms of getting to know athletes and hence the importance that coaches should promote personality screening and personality development programs.

Going into another of the pillars of this Research Topic, it is essential to study not only adult populations, but also the influence of all these factors on children and adolescents. Thus, Hao et al. show how in 126 students aged 4–5 years, functional physical training with or without cognitive intervention could promote physical fitness and cognitive development, this being a fundamental aspect when seeing not only physical, but also cognitive improvements in the population. In reference to somewhat older athletes, Haug et al. reflecting on more than 2,000 participants, the importance of motivation as a mediating role in understanding the factors related to Physical Education and cognitive development, considering the research results the importance of developing autonomous motivation to increase participation of adolescents in Physical Education classes. In the same paradigm, Tapan et al. show how adolescents and children must also work on technical and cognitive skills such as attention focus, especially when working on individual sports disciplines with an opponent such as tennis, showing, as in the case of tennis, instructions children to focus their attention externally to facilitate the learning of the groundstroke technique (forehand-backhand).

On the other hand, all research requires data analysis and in the science of psychology, the use of questionnaires is frequent, their validation being necessary to the context being worked on. The study by Su and Zhao allowed us to validate and develop a scale to measure the interpersonal style of coaches. The main findings of their research were that benevolent coaching behaviors held significant explanatory weight in the Chinese cultural context; controlling and autonomy-supportive coaching styles were culturally congruent among both Eastern and Western athletes; and benevolent and autonomy-supportive coaching behaviors positively impacted athletes, whereas controlling coaching behaviors had a negative impact. Finally, Wang et al. examined the properties of the Coach-Athlete Relationship Questionnaire in basketball players. This study supports the reliability and validity of this questionnaire, allowing the results to be extensible despite the existence of cultural differences in the place of validation of the initial questionnaire (Spain) and that of the study (China), being transcendental to contextualize all the variables when analyzed according to the place and objective of the study.

In conclusion, the work of every coach and teacher lies in ensuring that their athletes and students achieve adequate psychological strength in order to improve sports adherence and have a better quality in their sports life. Furthermore, in order to carry out studies, it is essential to have validated tools and diversify the study sample to generalize the results and seek the advancement of sports psychology.

## Author contributions

DM-S: Writing—review & editing, Writing—original draft. CB: Writing—review & editing. MA: Writing—review & editing. MG-L: Writing—review & editing, Writing—original draft.

